# Detecting Protein Candidate Fragments Using a Structural Alphabet Profile Comparison Approach

**DOI:** 10.1371/journal.pone.0080493

**Published:** 2013-11-26

**Authors:** Yimin Shen, Géraldine Picord, Frédéric Guyon, Pierre Tuffery

**Affiliations:** 1 INSERM, U973, MTi, Paris, France; 2 Univ Paris Diderot, Sorbonne Paris Cité, Paris, France; 3 RPBS, Paris, France; MRC National Institute for Medical Research, United Kingdom

## Abstract

Predicting accurate fragments from sequence has recently become a critical step for protein structure modeling, as protein fragment assembly techniques are presently among the most efficient approaches for de novo prediction. A key step in these approaches is, given the sequence of a protein to model, the identification of relevant fragments - candidate fragments - from a collection of the available 3D structures. These fragments can then be assembled to produce a model of the complete structure of the protein of interest. The search for candidate fragments is classically achieved by considering local sequence similarity using profile comparison, or threading approaches. In the present study, we introduce a new profile comparison approach that, instead of using amino acid profiles, is based on the use of predicted structural alphabet profiles, where structural alphabet profiles contain information related to the 3D local shapes associated with the sequences. We show that structural alphabet profile-profile comparison can be used efficiently to retrieve accurate structural fragments, and we introduce a fully new protocol for the detection of candidate fragments. It identifies fragments specific of each position of the sequence and of size varying between 6 and 27 amino-acids. We find it outperforms present state of the art approaches in terms (i) of the accuracy of the fragments identified, (ii) the rate of true positives identified, while having a high coverage score. We illustrate the relevance of the approach on complete target sets of the two previous *Critical Assessment of Techniques for Protein Structure Prediction* (CASP) rounds 9 and 10. A web server for the approach is freely available at http://bioserv.rpbs.univ-paris-diderot.fr/SAFrag.

## Introduction

Due to the large number of available protein structures and progress in remote homology detection, protein structure modeling is progressively focusing on smaller and smaller parts of protein structure [1]. Indeed, it has been suggested since several years that the diversity of the available structures may be sufficient to identify relevant templates for most of protein domains [2], and concomitantly, the number of new folds discovered has been decreasing constantly [3]. Protein fold identification being solved in most cases, fold adaptation under sequence divergence results however in local conformational modifications. Small or medium variations still remain to be addressed, in particular small variations in loops, or large insertion-deletions, as for instance recently illustrated in [4].

The search for short candidate fragments matching a sequence has been tackled within different contexts. Firstly there is interest in analyzing the structure/sequence relationship at the local level, as pioneered by Rooman and Wodak in the early 90′s[5]. Many studies have focused on identifying recurring patterns in protein structures, including among others, works related to structural alphabets (SA), fragment classification and many others [6]–[10]. Secondly, recurring sequence patterns have also been shown to correspond to conserved functional motifs [11]–[13], or conserved local structures, as demonstrated by the I-Sites of Bystroff and Baker [14], [15], again a motivation for many further studies. Finally, protein domains too divergent in sequence can remain out of the scope of homology modeling techniques, and it is interesting that progress in *de novo* modeling has also come from fragment assembly techniques - see for instance [16], [17], i.e. again considering the local conformation level. Hence, fragment identification has become a key step for both protein structure analysis, annotation and modeling.

The identification of candidate fragments starts from a given amino acid sequence. Different strategies have been described, considering fragments of fixed or variable length, considering gaps or not. I-sites [15] then nnmake [18] search for fragments of fixed length having a strong correlation between sequence and conformation so as to constrain the sampling of the conformational space to generate models. Approaches such as FRAGFOLD [19], TASSER [20], I-Tasser [21] or HHfrag [22] look instead for position-specific structural fragments. Profile comparison approaches have proven valuable to drive fragment identification. For instance, to identify fragments of variable lengths, HHfrag relies on the hhsearch Hidden Markov Model (HMM) profile comparison approach [23] - accepting gaps - adapted to short segments. Very recently, Xu et al. [24] have reported a gapless approach combining secondary structure, solvent accessibility, phi-psi angle prediction together with amino acid profiles derived from similar fragments from the PDB [25]. In the present study, we introduce a new approach to the detection of candidate structural fragments from the amino acid sequence. It is designed to search for candidate fragments of variable length at any position in the sequence. A major difference with former approaches that usually make use of enriched amino acid profiles, is that the search is based on predicted structural alphabet profiles.

A structural alphabet can be seen as a generalized secondary structure description allowing to label more accurately local conformations in the structures. Here, we use a Hidden Markov Model derived structural alphabet of 27 states - or structural alphabet letters - that describes the proteins as series of overlapping fragments of 4 amino acid length [7]. The HMM model consists in multivariate gaussians describing the specific geometry of each letter and the transition matrix associated with the Markovian process. Given such model and a protein structure, classical HMM techniques can infer the optimal states at each position, which allows to translate the 3D coordinates of a structure of 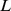 amino acids as a series of 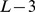 letters of the structural alphabet. Starting from an amino acid sequence turns into a prediction problem. Using a machine learning approach, we have previously setup an approach that predicts the probabilities of the structural alphabet states from an amino acid sequence. Given a sequence, it returns, for each fragment of 4 amino acid length in the sequence, the probabilities that it is associated with each of the structural alphabet states. Thus, given a sequence of size 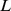, it returns a series of 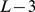 profiles of dimension 27, which we call here the *predicted structural alphabet profile*. In previous studies, we have shown such prediction can be used to enhance protein domain fold attribution from sequence compared to other approaches using features derived from amino acid profiles [26]. We have also designed PEP-FOLD, a *de novo* approach to peptide modeling [27] based on such prediction. PEP-FOLD selects, at each position in a peptide sequence, the n-best letters - 8 in the current version, so as to limit the sampling of the local conformational space to perform the 3D assembly. PEP-FOLD results have shown that the truncated profiles are efficient to grab information about the local structure. Here, we turn to the full - not truncated - comparison of the predicted structural alphabet profiles of dimension 27. We investigate how profile comparison can be applied to the search for candidate structural fragments. We introduce a new approach based on such profile comparison. We find it outperforms state of the art profile-profile approach such as HHfrag [22] in terms of specificity and precision, while reaching a coverage of the targets close to 90%.

## Results

**Figure 1:**
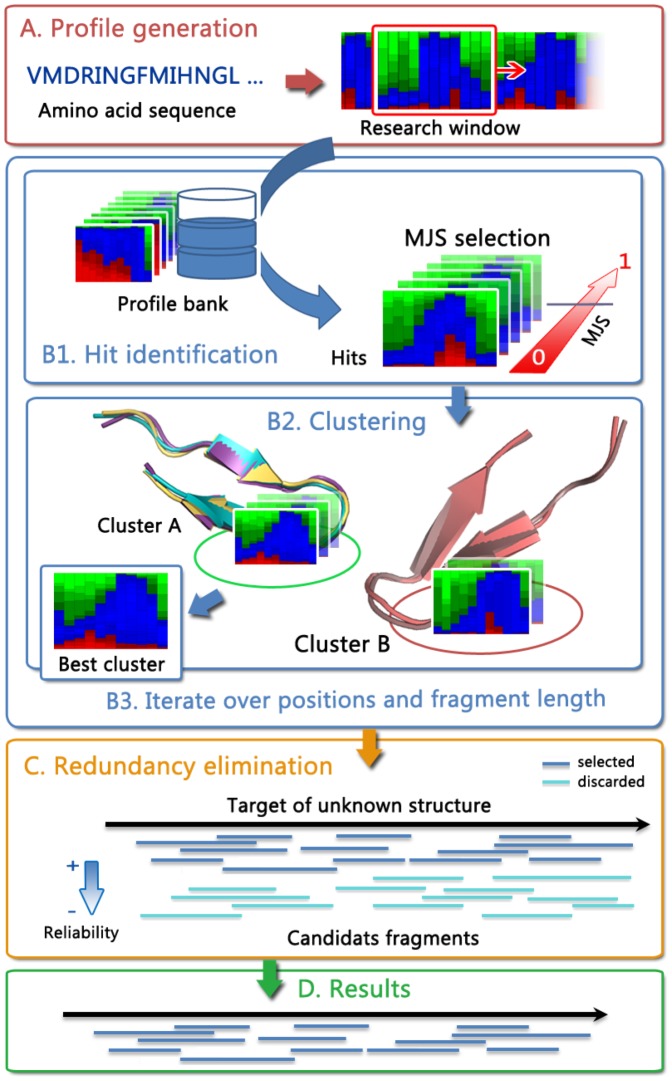
The SA-Frag protocol. A: Structural alphabet profile prediction from amino acid sequence. B1: Fragment search using profile comparison against a bank of predicted profiles. B2: Clustering to discard spurious matches. C: Hit redundancy elimination.

We have developed a new protocol to identify candidate structural fragments given the amino acid sequence of a query. At each position of the sequence, it searches for candidate structural fragments of variable length by mining a bank of structures. The search is performed for each query sub-sequences of size between 6 and 27 amino acids, presently using a brute force strategy: for each sub-sequence, a systematic scan of all proteins in the bank is performed, sliding the sub-sequence at each position of each protein, not allowing gap, and scoring the similarity. The scoring is based on the comparison of the structural alphabet profile of the sub-sequence with that of the protein scanned, using the Jensen Shannon divergence. A flowchart of the complete approach is presented Figure 1. It consists in three steps. The first one is the generation of a structural alphabet profile given a query sequence. The second one is the systematic search for matches mining a collection of pre-generated profiles for proteins of known structure, and the identification of hits, i.e. matches having a score better than a given value. Since the number of hits is potentially very large, several mechanisms have been implemented to limit their number (see methods). The most important consists in the clustering of the hits to identify the most relevant candidate fragments. Also, since we consider various fragment size at each position, it is possible that nested matches are identified. Consequently, the third step performs some redundancy elimination over the hits of different sizes collected. The underlying strategy of this last step is to sort the matches according to their expected precision, and to favor hits having the best expected precision, while preserving protein coverage by accurate fragments (see methods).

### Low profile-profile distances to identify similar fragments

We first assess the effectiveness of the profile-profile scoring to identify candidate fragments. The profiles correspond to the predicted probabilities that the sequence of each fragment of 4 amino acids adopts one of the conformations of the structural alphabet. The distance between two profiles is measured using the maximum Jensen Shannon (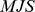) criterion (see methods). Low alpha carbon RMD deviation (cRMSD) matches are expected to have low 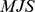 values.

**Figure 2:**
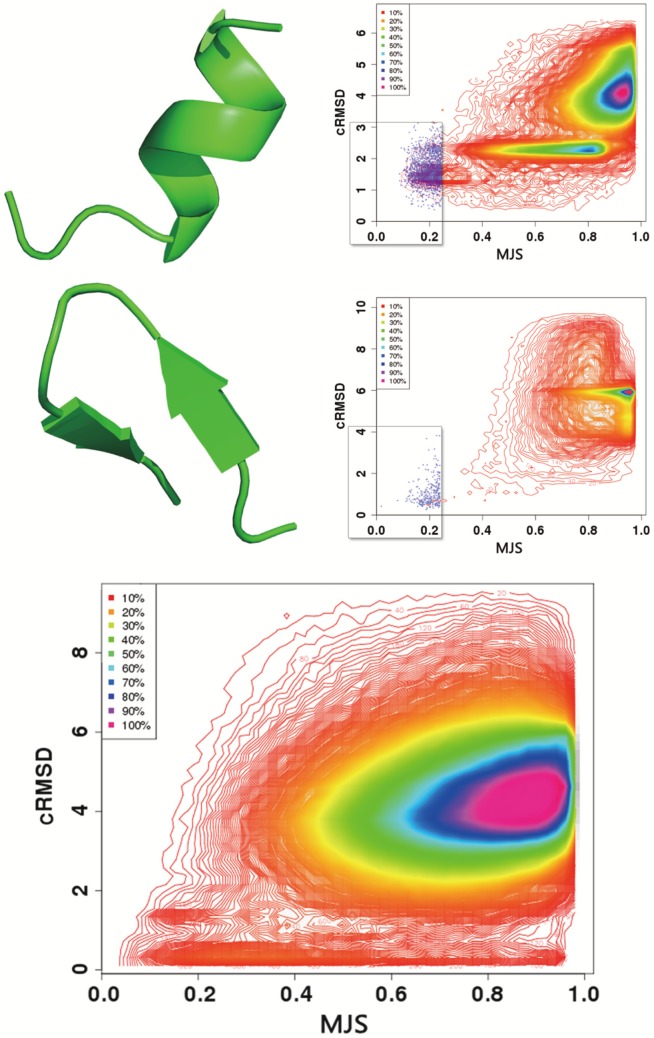
Relationship between the alpha carbon RMSD (cRMSD) and the profile similarity score (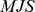). Top: PDB entry 3no6, residues 143–153. Middle: PDB entry 3nmb, residues 84–94. The fragments are matched against all the fragments of size 11 amino acids of the PDB25 set. Bottom: Values collected for 799 fragments of eleven amino acids and of different conformations are depicted. Over 500 000 pairs are depicted. Iso contour representation is used, except for the areas of low cRMSD and low 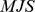 - surrounded by a rectangle - for which individual dots are depicted.

Figure 2 shows the distribution of the 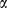-carbon RMSD (cRMSD) as a function of the 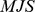 for two fragments of eleven amino acids adopting helical conformation - CASP9 target T0516, PDB entry 3no6, fragment 143–153, and a beta hairpin conformation - CASP9 target T0518, PDB entry 3nmb, residues 84–94. The plots depict the complete distribution of the 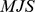 and cRMSD values obtained over the complete PDB25 dataset, i.e. over 637 000 comparisons. For sake of clarity, only the iso contours of the densities are plotted, except for the regions of low 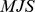 and low cRMSD - areas surrounded by a rectangle - for which the individual dots are plotted.

A first observation is the apparent difference between the distributions. Three regions of stronger densities are observed for the helical fragment and only one for the beta hairpin. In fact the two plots are consistent. Firstly and non surprisingly, in both cases, the areas with the strongest densities occur for simultaneously large cRMSD and MJS values (yellow to purple areas) and correspond to irrelevant matches. For the alpha helical fragment, an additional area of strong density is observed for cRMSD close to 2Å. It corresponds to the alpha helices of the dataset, the 2Å deviation coming from the non helical C-terminus of the query. The third region observed for cRMSD close to 1Å corresponds to the true hits. Its absence for the beta hairpin is only apparent and results from a threshold effect in the iso contours, as can be seen looking at individual dots in the regions of low MJS and low cRMSD. Compared to a helical fragment, fewer hits are expected for a beta hairpin because the larger conformational stability of a helical fragment compared to that of a beta hairpin makes the expected number of very low cRMSD fragments larger for helical conformations. Thus, for both cases, low 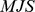 values are associated with low cRMSDs, which implies the predicted profiles can lead to the identification of relevant fragments.

**Figure 3:**
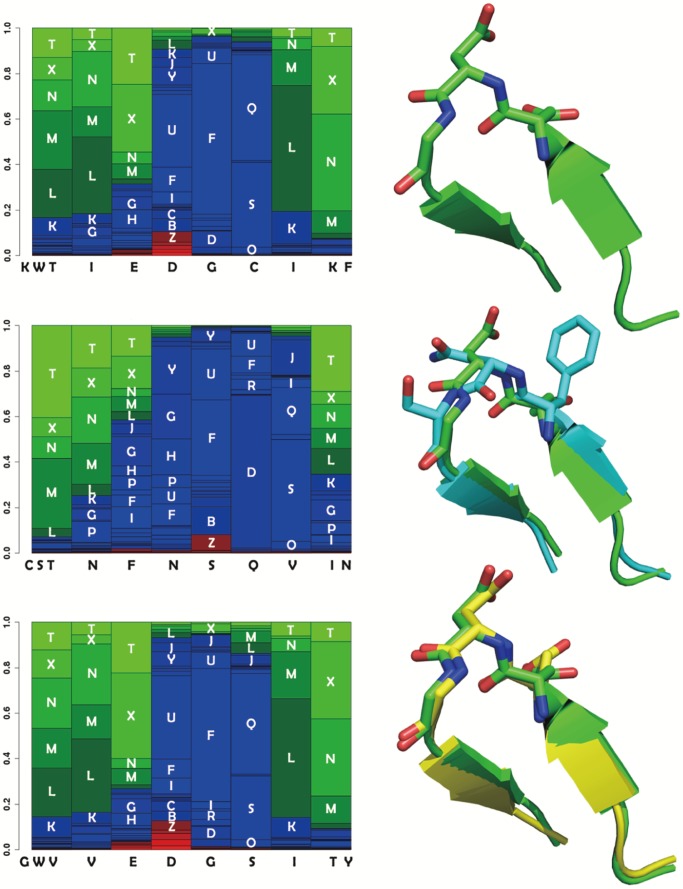
Examples of profile similarity score variation for low cRMSD fragments. For three fragments, their structural alphabet profiles (left) and their structure (right) are plotted. The x axis of the profiles corresponds to the amino acid sequence. The y axis corresponds to the predicted probabilities of each of the 27 local conformations of the structural alphabet. For sake of clarity, the conformations are sorted from the most helical (red - bottom) to the most extended (green - top). Blue conformations correspond to coil. Each column corresponds to a fragment of 4 amino acids. For instance, the first barplot of the upper profile corresponds to the amino acid sequence KWTI, the second to WTIE, etc. The local conformation labels associated with sufficiently large probabilities are printed as overlays of the profile. The two first positions of the upper profile - corresponding to KWTI and WTIE - have large probabilities for extended conformations (labels T,X,N, M and L). Top: PDB entry 3nmb, residues 84–94. Middle: PDB entry 1xau chain A residues 95–105 (blue), superimposed on the 3nmb fragment (green). The profiles differ at positions 6 and 7. At position 6, the largest probability is associated with local conformation label Q for 3nmb and D for 1xau. At position 7, the largest probability is for conformation label L for 3nmb and S for 1xau. Slight differences are observed between the backbone conformation of the two fragments observed. The 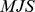 score between the 3nmb and 1xau fragments is of 0.77 for a cRMSD of 0.71. Bottom: PDB entry 3h3l chain A, residues 75–85 (yellow), superimposed on the 3nmb fragment (green). The 3h3l fragment profile is very similar to that of the 3nmb fragment. The 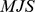 score is of 0.02 for a cRMSD of 0.43.

A second observation is that a rather large range of 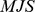 values can be associated with low cRMSDs. For instance, for the beta hairpin, fragments having a cRMSD less than 2Å have 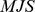 values between 0.1 and 0.9. Figure 2-Bottom depicts the relationship between the cRMSD and the 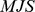 collected for close to 800 fragments of eleven amino acids. It contains over 500 000 dots. A similar behavior is observed. One such example of profile mismatch for low cRMSD is detailled Figure 3. One fragment (PDB 3h3lA: 75–85) (bottom) has a profile very similar to that of the query (PDB 3nmb:84–94) (top) - distance of 0.11, and the conformations are very close - cRMSD of 0.44Å. For another fragment (PDB 1xauA: 95–105) (middle) the profile differs on its C-terminus end. Particularly, at the penultimate position, the large frequency for letter L is superseded by a large frequency for the letter S, and the profile distance at this position is of 0.77 for a cRMSD of 0.72Å. We have investigated more in detail the reasons why such large panel of 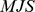 values are associated with lows cRMSDs and we have found several reasons can explain it. Firstly, the distance criterion presently in use measures the similarity by pairing the probabilities of identical local conformations. It does not consider the possibility for equivalences between non identical conformations, which is over restrictive. Indeed, the analysis of collections of structural alignments has shown some equivalences between the structural alphabet letters exist, an observation that has been used to derive structural alphabets similarity matrices used for structural alignment techniques [28], [29]. Consequently, some part of the divergence between profiles can be attributed to such equivalence between similar 3D shapes. It turns however that considering such equivalences in the distance between profiles adds some complexity and it remains the subject for further work. Secondly, it is possible that differences in profiles are the result of errors in the prediction, even if our previous analyses related to the 3D *de novo* modeling of peptides [27] suggest full misprediction of the local conformation is only marginal. A third explanation is that the cRMSD criterion itself can be misleading [30]. Low cRMSD fragments can include local deformations that will correspond to different local conformations as assigned by the structural alphabet. Thus for fragments of low cRMSDs, it is possible that a correct prediction can lead to large 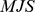 values.

**Figure 4:**
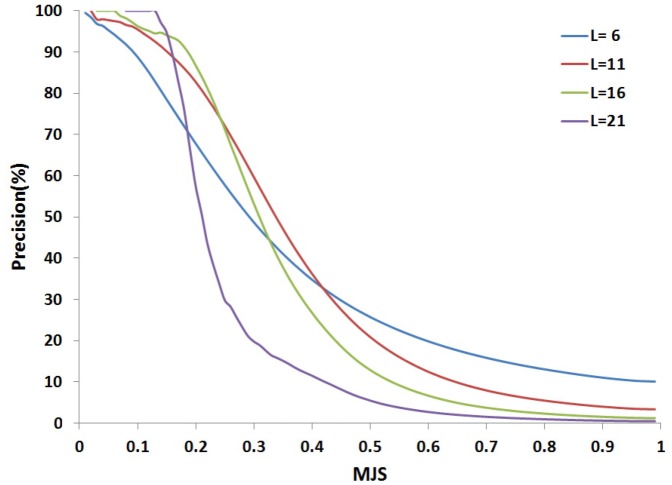
Precision as a function of the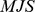. The precision is estimated for fragments collected for a given MJS threshold. For each fragment size, curves are calculated from over 5 000 000 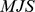/ cRMSD values obtained by scanning the PDB25 set with fragments of the CASP8 targets.

Since it is hard to assess the exact amount of cases that could be attributed to each of the aforementioned explanations for having fragments of low cRMSDs but large 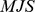 values, the sensitivity of a procedure using the 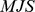 to identify candidate fragments is necessarily underestimated. However, it is important to note a possibly weak sensitivity is not necessarily too penalizing. Firstly, it can be compensated by the very large number of comparisons achieved mining large collections of structures for different fragment sizes. Secondly, the main objective of the approach is to reach a good specificity, i.e. identify fragments that are accurate and reject inaccurate ones, so as to propose correct fragments for 3D modeling. Interestingly, as depicted Figure 4, a clear relationship exists between the 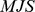 value and the precision of the fragments collected. In practice, for each fragment size, we have used the 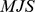 value for which the precision is of 0.95 as the threshold to identify hits.

### Clustering as a mean to infer an expected precision

We now consider a second important condition for efficient structural fragment identification. The approach should return as few erroneous fragments as possible, where erroneous means a fragment adopting an incorrect conformation that will lead to poor modeling. This can be measured as the precision of the approach, i.e. the ratio of the *correct* fragments upon all fragments returned, that should be as high as possible. Consequently, means to control the expected precision of the protocol are important. To this respect, looking at Figure 2, a last observation of interest is that clusters are observed in the region of low 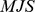 of plots A and B. Such clusters are associated with low cRMSDs, outliers having larger cRMSD values. For instance, the cRMSDs of the hits at 0.2 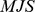 for the beta hairpin vary between 0.1 and 4Å, but a larger density of hits is obtained for 0.2Å. This suggests that clustering the hits and analyzing cluster effectives could be a mean to enforce true positive hit identification. To assess the generality of this observation, we have performed a large scale analysis collecting, for all fragments of size between 6 and 27 amino acids of all CAPS8 targets, information about the clusters returned by step two of our protocol, using the PDB25 set. Each cluster has been associated with three informations: its effective, the MJS value associated with its centroid, and the quality of the centroid in terms of true positive or false positive, according to the cRMSD between the query and the centroid. Finally, we have analyzed the relationship between the cluster effectives (Weight - W), the 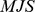 score, and the *a posteriori* precision.

**Figure 5:**
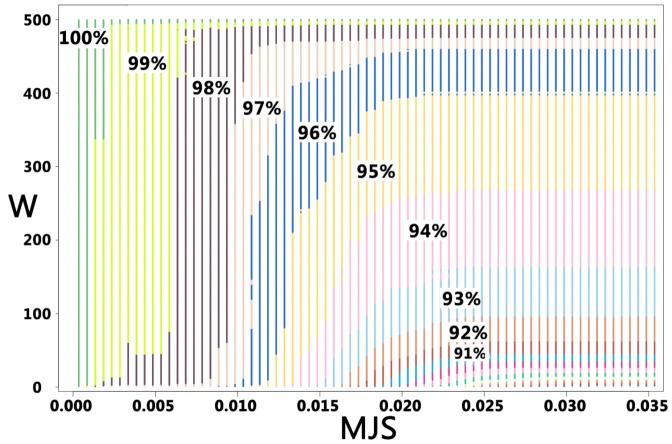
Cluster effectives(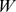) and 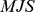 score. The plot contains all the clusters identified for all the fragments of 9 amino acids of the CASP8 targets against the PDB25 set - over 15,100 clusters. Each cluster is associated with the 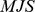 value of its centroid, and its effectives (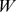). For each area of the plot colors are used to depict the cumulative precision of the clusters - labels in %. Steps of 0.001 have been used for the x axis (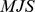) and 1 for the y axis (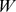). Clearly delimited regions are observed, which allows to infer an expected precision given the 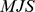 and 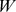 values of a cluster.

Figure 5 shows for fragments of size 9 amino acids, the relationship between the effectives of the clusters (Weight - W), and the 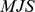 score of their centroids. The plot contains over 15,100 clusters. For each area of the plot, we have calculated the cumulative observed precision, i.e. the precision over all clusters with a 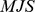 less than and of 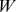 more than the current values, using steps of 0.001 for 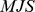 and 1 for 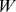. Different colors are used to depict the cumulative observed precision (labels in %). Clearly, it is possible to associate areas of the plot to an expected precision, which provides a simple mean to infer the expected precision of a cluster given its weight and 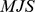 score. In addition, the observed precision is large for low scores. It is of over 95% for scores less than 0.15. Thus, clusters with high effectives collected using the 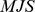 score are associated with high values of observed precision. We have performed similar analyses for each fragment size, and for each, we have identified an expected precision as a function of the cluster effectives and 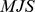 score. These values are in use in the final redundancy elimination procedure of our protocol (see methods).

### Fragment identification

**Figure 6:**
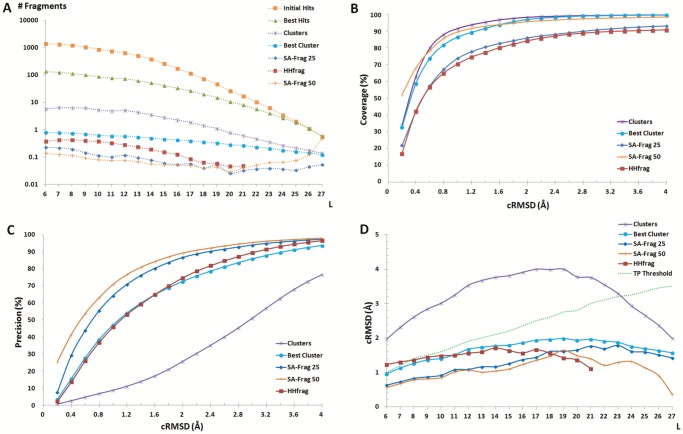
SA-Frag performance at different steps of the search. A: average number of hits returned per request for fragment size varying between 6 and 27 amino acids. B: coverage, the fraction of amino acids covered by at least one candidate fragment, C: precision, the ratio of correct candidates over the total number of candidates. D: average cRMSD of hits as a function of fragment size (L). The different lines correspond to different steps of the protocol (see methods). HHfrag results are also reported. All curves but the one labeled SA-Frag 50 (obtained using the PDB50 set) are obtained using the PDB25 set.

The analysis of the performance of the complete protocol is summarized in Figure 6. We first look at the performance obtained over all CASP9 targets, mining a subset of the Protein Data Bank filtered at 25% sequence identity (PDB25), i.e. in conditions of low sequence homology. Note this dataset was built in April 2010, i.e. before CASP9. Figure 6A shows the evolution of the number of hits at the different steps of the protocol (see methods). For sake of clarity, we report the number of hits in terms of the number of hits per elementary search, i.e. averaged over the different sequence indexes and different fragment sizes. One sees the initial number of hits is very large. It is of over 1000 collected fragments per request for fragments of 6 amino acid length. Although fewer fragments are collected for larger sizes, occurrences of similar larger fragments being less likely to occur, it is by far too large to be handled over a complete sequence. Interestingly, the consecutive filters (best hits, clusters, best cluster and redundancy elimination) allow to reduce this number by close to one order of magnitude each, which, on average, reduces the final number of hit per request to a value close to only 0.1. As a result, the mean number of fragments returned is on the order of only 160 for a protein of 100 amino acids.

Figure 6B presents an analysis of the coverage (percentage of the query residues covered by at least one hit). One observes that the value of the coverage obtained during the initial steps of the protocol is over 90%, while the final redundancy elimination procedure has a large impact, a decrease by over 15% for 1.2Å. Since this procedure makes use of the expected precision of the fragments to eliminate redundancies, one can expect that most of the fragments discarded are of poor quality. Actually, Figure 6C shows that the impact of this final step is effectively to increase the precision of the hits. Consistently, Figure 6D shows that the successive steps of the procedure largely improve the average accuracy of the hits and result in cRMSDs much below the limit of significance (dashed line). On average, most of the fragments are associated with cRMSDs of less than 1.5Å. In summary, the different steps of the protocol allow to dramatically reduce the number of candidates - by close to 4 orders of magnitude, while increasing the precision of the hits and preserving a coverage of 86% for a fragment accuracy better than or equal to 2Å cRMSD.

To investigate if the protocol can take advantage of mining closer homologs, we have also performed a similar experiment using a subset of the Protein Data Bank filtered at 50% sequence identity (PDB50). Figure 6 only reports the final results after the complete protocol. One sees mining the PDB50 set instead of the PDB25 set does not result in a number of hits significantly larger - Figure 6A. One observes an important gain in coverage (over to 95% for 2Å cRMSD) - Figure 6B. It is associated with a slightly increased precision - Figure 6C-, and an accuracy of the fragment unaffected, except for large fragment sizes where one observes an increase in the number of hits and a decrease of their average cRMSD. This indicates that homologous long hits are better identified. Overall, such results suggest the main outcome of using the procedure on homologous structures is not in terms of increased accuracy and precision, but in terms of coverage. Larger parts of the structures are covered with accurate fragments.

### Comparison with previous approaches

**Figure 7:**
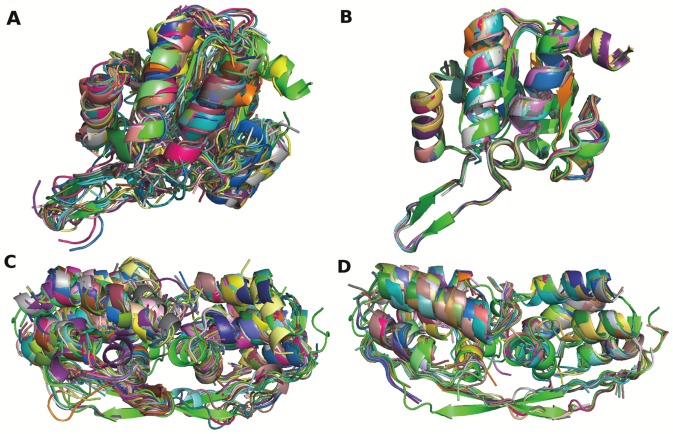
Candidate fragments obtained using HHfrag (A,C) and SA-Frag (B,D) for CASP9 target T0635 - Template based (A,B) and CASP10 target T0734 - Free Modeling (C,D). The search was performed using the PDB25 set. The fragments are superimposed on the experimental structure (green).

The added value of using structural alphabet profiles can be discussed by comparing our procedure (SA-Frag) with HHfrag, an approach based on amino acid profile comparison recently reported to outperform other approaches such as nnmake [18] or torusDBN [9]. We first compare HHfrag and SA-Frag for the CASP9 targets, using the PDB25 protein collection - Figure 6. Overall, the two approaches reach very similar performances in terms of coverage. One observes a slightly more even distribution of the hits depending on size for SA-Frag (Figure 6 A). A major difference is observed for the precision of the fragments, where the precision reached by SA-Frag is much larger. At 2 Å cRMSD, SA-Frag precision is of 86.7% and that of HHfrag of 74.7%, i.e. a gain of close to 12%. Consequently, the accuracy of the fragments collected by SA-Frag is usually better than those identified by HHfrag, up to fragment size of 18 amino acids - Figure 6 D. This performance is obtained for a number of fragments that is smaller than that of HHfrag - Figure 6 A. Over all targets, the average number of fragments identified is of 686 for HHfrag, when it is only of 349 using our approach. Hence, as illustrated for two targets Figure 7, using SA profiles results in better quality fragments. The final performance of the two approaches are summarized Table 1. Over the complete CASP9 target set, SA-Frag outperforms HHfrag in terms of coverage (Cov), coverage considering only true positives (TPCov), and in terms of precision for which differences of 4, 4 and 19% are observed, respectively. Looking more in detail at the more difficult Free Modeling domains, one observes, as could be expected, a decrease in performance for both approaches. However, compared to the results obtained over the complete target set, the precision of the fragments collected by SA-Frag is only moderately affected compared to HHfrag.

**Table 1:** HHfrag and SA-Frag performance for CASP9 and CASP10 targets.

	CASP9		CASP10			
All targets	HHfrag	SA-Frag	HHfrag	SA-Frag	HHfrag50	SA-Frag50
Cov.	91.0	94.6	83.6	93.8	92.5	99.6
TPCov.	78.2	82.7	71.2	81.8	84.2	90.9
Prec.	65.9	84.7	69.8	83.9	68.7	85.4
FM domains						
Cov.	84.9	88.5	89.4	92.5	97.0	99.8
TPCov.	61.9	71.4	72.1	75.3	82.8	85.7
Prec.	50.2	80.0	50.6	80.2	52.5	80.5

HHfrag and SA-Frag and HHfrag (resp. SA-Frag 50, HHfrag50) results were obtained using the PDB25 (resp. PDB50) set. Cov.: the fraction of amino acids of the target covered by at least one candidate fragment. TPCov.: the fraction of amino acids of the target covered by at least one accurate candidate fragment (True positive). Prec.: The fraction of True positive candidate fragments among the candidate fragments returned.

We have also assessed the relative performance of both approaches on the very recent CASP10 target set. Considering all the targets, the performance of both approaches appears rather stable over CASP9 and CASP10 target sets. As for the CASP9 targets, Table 1 shows that the SA-Frag performance is better in terms of coverage (Cov), coverage considering only true positives (TPCov), and in terms of precision. Using the PDB25 set on the complete target set, the improvement is of close to 10%, 10% and by over 13%, respectively. For the FM domains, the results appear a bit better for CASP10 than for CASP9, suggesting that the difficulty of the targets was greater for CASP9, but overall, the results are consistent. Moving from PDB25 to PDB50, the same trends are observed, but the TPCov of both SA-Frag and HHfrag is largely improved, by close to 10%, reaching over 90% for SA-Frag. The precision of the two approaches appears stable compared to the results using PDB25. Investigating more in details the performance for the more difficult 14 Free Modeling domains of CASP10, we find that, compared to the complete target set, HHfrag results are similar in terms of coverage, but for a degraded precision, whereas SA-Frag TP coverage is degraded for a precision moderately affected. We find consistent results using the PDB50 set, where both HHfrag and SA-Frag TPCov are increased by close to 10% for a precision similar to that obtained with PDB25.

Finally, we have also considered combining the hits returned by HHfrag and SA-Frag. The results are reported Table 2. The results obtained for CASP9 and CASP10 targets appearing similar, we discuss in detail the results obtained for CASP10 targets. Using the PDB25 set, we obtain for the complete CASP10 target set, a TPCov value of 87.7%, i.e. a gain of 15% and 6% compared with HHfrag and SA-Frag alone, respectively. This clearly suggests that each approach is able to address specific but distinct aspect of hit identification. An improvement is also observed for FM domains, of 12% and 9%, respectively. Using the PDB50 set, the coverage by true positive fragments reaches 94.4% over all CASP10 targets, and 90% on the FM domains, the best results of this study. However, this increase is at the cost of a decrease of the precision that is as low as 70.6% and 61.7% considering all target and FM targets, respectively. We have analyzed the distribution of the specific contributions in terms of secondary structure. Using the PDB25 set, we find for SA-Frag that the fraction of amino acids covered by TPCov is of 93.2, 84.0 and 72.4% for alpha, beta and coil residues. It is of 82.1, 76.0 and 57.2% for HHfrag. The largest difference is observed for coil residues. The fraction of sites not covered by a TP hit using HHfrag for which SA-Frag finds a TP hit is of 17% - it is of 5% conversely. A more detailed analysis show that, starting from HHfrag results, the fractions of alpha, beta and coil sites recovered by SA-Frag are of 26.6, 18.7 and 54.6%, respectively. Corresponding fractions for HHfrag enhancement over SA-Frag results are of 16.7, 26.4 and 56.7%. Hence, most of the improvement combining the approaches arises for coil residues. The same trends are observed for the FM targets.

**Table 2:** Combined HHfrag and SA-Frag performance for CASP9 and CASP10 targets.

	CASP9	CASP10	
All targets	HHfrag + SA-Frag	HHfrag + SA-Frag	HHfrag50 + SA-Frag50
Cov.	98.8	98.4	99.9
PCov.	88.6	87.7	94.9
Prec.	72.2	74.6	70.5
FM domains			
Cov.	96.8	98.2	98.6
PCov.	76.4	84.7	90.7
Prec.	63.6	64.7	61.7

Results obtained when merging the candidate fragments identified by HHfrag and SA-Frag. HHfrag and SA-Frag and HHfrag (resp. SA-Frag 50, HHfrag50) results were obtained using the PDB25 (resp. PDB50) set. Cov.: the fraction of amino acids of the target covered by at least one candidate fragment. TPCov.: the fraction of amino acids of the target covered by at least one accurate candidate fragment (True positive). Prec.: The fraction of True positive candidate fragments among the candidate fragments returned.

## Discussion and Perspectives

In the present study, we have introduced a new approach for the identification of candidate fragments, based on the comparison of structural alphabet profiles predicted from the amino acid sequence.

As a more detailed characterization of the local structure of proteins compared to the classical secondary structure conformations [31], structural alphabets have previously proven efficient for the analysis of protein conformations, to identify and align similar structures [28], [32], to quantify the statistical significance of structural motifs [13], or to track conformational changes through ensembles of conformations resulting from simulated annealing or molecular dynamics simulations [33]–[36]. Such analyses have relied on the identification of only the most representative local conformation at each position in a conformation. However, sub-optimal encoding can be expected to contain information as well since each state is associated with a variability and since it is well known that some equivalences between the local conformations can be inferred from structural alignments. The need to consider suboptimal conformations is even greater when using local conformations predicted from an amino-acid sequence. Probability profiles are usually much smoother than those obtained from 3D structures, and the optimal conformation can be ranked at a sub-optimal position [27], [37]. This can be attributed for one part to a weak sequence structure relationship for some sequences or some parts of sequences. Interestingly, it also seems such profiles can embed information related to the intrinsic flexibility of protein structure as a function of their entropy [38], suggesting several conformations can be associated with one local sequence. It thus seems highly desirable to explore ways considering not solely the best conformation predicted but also the other ones, as proven for instance during the design of the PEP-FOLD *de novo* prediction approach[27], [39]. Ultimately, all the probabilities of the local conformations at each position in a structure could be informative. The present study is, to our knowledge the first attempt to use the complete information of predicted profiles of local conformation, and our results clearly indicate that such predicted structural alphabet profiles are a relevant mean to grab sequence-conformation relationships at a local level. Indeed our results show that, compared to previous approaches, the major benefit of such approach is its ability to identify fewer hits, but at a better accuracy and precision.

Among the possible reasons for such improvement, the main one is that predicted structural alphabet profiles directly code for local conformation information, a difference with amino acid profiles. Since structure is known to be more conserved that sequence, such encoding could be more relevant, and our previous results about fold attribution [26] support this idea. In a general manner, the consideration of structural features has been shown to supplement efficiently pure amino acid information. It is for example the case for the approach described by Xu and Zhang [24] that, even if more oriented towards contact prediction, makes use of profiles combining amino acid frequencies and predicted structural features.

Another explanation could be that the higher dimension of the profile - 27 here - could help to grab some accurate details of the local sequence - structure relationship. Following, one could ask how the choice of the collection of canonical local conformations impacts the results. Interestingly, the protocol described here is not specific of the particular structural alphabet used, and indeed, numerous collections of short fragments - denominated as structural alphabets or not - have been described by other groups (e.g. [6], [8], [9], [40], [41] among many others). However, they vary largely both in the descriptors and methods used to identify them, and consequently in size. All these differences make likely that the transposition of the protocol to other structural alphabets would require slight but specific adaptations for each step of the protocol.

Turning back to the present procedure, our goal here was primarily to assess the effectiveness of the concept. It is clear it could be improved on several aspects. It is obvious that the brute force strategy used could be revisited. Redundant Jensen-Shannon calculations are presently performed which could be optimized. As well, the use of the Jensen-Shannon divergence instead of other criterion such as the Skew divergence could be questioned. Following, as discussed before, the weak sensitivity of the protocol could also be the matter for further developments, in particular accepting some equivalence between local conformations. Such question is clearly of theoretical interest although preliminary tests show it is difficult to foresee the balance between the gain in sensitivity and a possible loss in precision when accepting equivalence between the local conformations. In terms of candidate fragment identification however, present results clearly show that the large number of structures available already allows to identify accurate fragments for over 90% of a query sequence. Finally, it could be even more interesting to combine two approaches such as HHfrag and SA-Frag, two profile comparison approaches - although of different kind, to bring a significant improvement. Embedding SA profiles in the HMM profile comparison approach of HHfrag could be a promising perspective. Despite this would open the door to similarity search at larger scale, this perspective still faces however the difficult question of efficiently modeling gaps in structural alphabet profile comparison.

## Materials and Methods

### Datasets

We have considered five different datasets. The first one (PDB25) corresponds to the 4 678 proteins used by [22] to assess HHfrag performance, reduced to 4 649 after removing fragmented structures. A second one (PDB50) corresponds to the 10 114 proteins identified by the culled PDB [42] of April 13th, 2012, at less than 50% sequence identity, a resolution better than 2Å and a R-value less than 0.25. The three other sets correspond to the targets of the three last CASP editions, and correspond to 120 proteins for CASP8, 111 for CASP9 and 96 CASP10 targets for which experimental coordinates are available, including 25 and 14 free modeling (FM) domains for CASP9 and CASP10, respectively. For the HHfrag experiments, the hhm profiles of the sequences corresponding to the PDB25 collection have been kindly provided by the HHfrag authors [22]. The hhm profiles of the sequences corresponding to the PDB50 collection have been generated using HHblits with the nr20 databank and hhmake of the hhsuite package [1].

### Generation of the SA-profiles

We describe the protein structures using a Hidden Markov Model derived structural alphabet of 27 letters [7]. In this framework, protein structures are considered as series of fragments of 4 amino acids overlapping by three residues. Given a protein structure of size 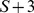, it is possible from local geometrical descriptors to identify the optimal series of size 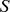 of the letters that describe it using the Viterbi algorithm, or to assess the probability that each letter represents the local conformation at each position of a structure using the forward-backward algorithm, which results in a profile of size 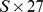. The way the SA has been learnt, the discussion about its optimality, the description of the SA letters and the values of the descriptors associated with them can be found in [7].

Starting from an amino acid sequence, the SA-profiles are predicted using a protocol identical to that described in [27]. Briefly, the prediction is performed using a support vector machine taking as input a 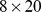 vector that correspond to the PSI-blast profile of a segment of 8 amino acids, where the 4 central amino acids correspond to a structural alphabet letter, and two residues are added each side. The SVM in use in this study has been learned on a collection of 3672 protein structures resolved earlier than 2006. Updates of this collection have so far not led to significant improvement, which can be related to the locality of the prediction. The output is a vector of size 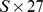 that corresponds to the predicted SA profile.

### SA profile comparison

The comparison of the predicted SA profiles is performed assuming an ungapped procedure. In our experience [28] and those of others [43], the management of gaps in the structural alphabet framework can be misleading as a single insertion or deletion may lead to a major change in the local conformation of the backbone. So far, instead of considering HMM-HMM alignment techniques such as embedded in HHpred or HHfrag for instance [22], [23], we use a more straight approach based on the Jensen Shannon distance to compare two SA profiles 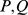 of dimension 27:
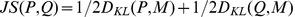
(1)


where M is 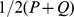 and 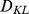 is the Kullback-Leibler divergence
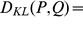
(2)


where P(i) is the probability of SA letter 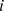.

For two vectors of profiles of size 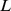 - corresponding to the profiles describing two fragments of size 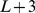 amino acids, we use as distance:
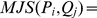
(3)


where 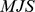 stands for the Maximum Jensen-Shannon over the paired series of profiles, 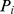 and 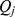 are the two profiles corresponding to positions from 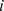 to 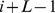 on the first sequence and 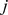 to 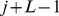 on the second one. Note that a 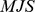 distance of 0 indicates a perfect identity of the profiles. Such case could occurr when mining a bank containing the exact same protein sequence since the profiles would be identical, but in theory, it is also possible that two profiles are identical for non identical structures since (i) prediction could in theory produce identical profiles for different shapes, and (ii) identical series in the structural alphabet space encompass structural fuzziness - see [7].

### Significant fragment identification

Given a query of size 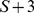, the search for hits is achieved considering profiles of length 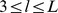, where L has presently a value of 24, over positions 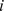, where 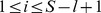. Each fragment is paired with all fragments of equal size in the 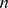 proteins of the bank. For each fragment pair, SA-Frag measures the 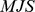 distance. Only the fragments that have a value of 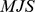 lower than a given threshold are selected. The 22 - fragment size from 6 to 27 amino acids - thresholds used for this study have been learnt over the complete collection of 120 CASP8 targets. A control over the 111 CASP9 targets using a 5 fold cross validation procedure resulted in threshold values only slightly different, which suggests the threshold values are rather independent from the learning set.

Since the number of hits can be very large (for instance several millions of hits can be selected for a short helical fragment), the search procedure embeds two mechanisms to limit their number. Firstly, for each query, we limit the number of hits to the first N best hits, in practice 500. Secondly, the N best hits are sorted according to their 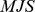 scores and clustered by an incremental procedure using the alpha carbon RMS deviation (cRMSD) of the fragments as a score of similarity. The cRMSD thresholds used for clustering depend on fragment length. The goal is to select only the classes with the highest densities, those of low densities being expected to correspond to noise. Class representatives are incrementally defined as the fragments having the lowest 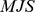 values, and the effectives of the class (or weight - 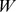) are kept in memory. After this step, only one fragment - the cluster representative - is selected per cluster. This allows a dramatic reduction of the number of hits. Finally, we have also found from a posterior analysis (see Results) that the observed precision is higher for large values of 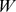. Thus, for each query, only the cluster with the largest 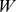 value is kept. If several clusters have identical 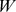 values, the one which has the lowest 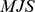 value is retained.

A last filtering step is achieved by considering the expected precision of the candidate fragments. All hits associated with an expected precision more than an upper threshold are kept, and those with an expected precision less than a lower threshold are discarded. Fragments having an expected precision in between these two thresholds are used as best candidates for regions not yet covered. Since these fragments are associated with a less predictable quality, we accept some redundancy in these regions. In practice, it is possible to adjust the values depending on the desired ratio between coverage and precision. In this study, we used upper and lower values of 0.99 and 0.82 (resp. 0.995 and 0.65) with the PDB25 (resp. PDB50) set. Nested hits from the same protein are discarded.

### Assessment of fragment quality

To assess the results, we define true positives (TP) the hits deviating from the query by less than a given cRMSD. Since the distribution of the cRMSD over random fragments depends on fragment size, we follow the rule used in [22]. TPs correspond to fragments with a cRMSD less than 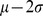 where 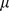 is, for a given size, the average cRMSD over the whole dataset, and 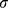 the associated standard deviation. Other hits correspond to false positives (FP). The precision is then defined as: 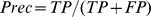. The coverage (Cov) is the percentage of target residues that are covered by at least one hit. The true positive coverage (TPCov) is the percentage of target residues that are covered by at least one TP.
